# Dietary Modification for Reproductive Health in Women With Polycystic Ovary Syndrome: A Systematic Review and Meta-Analysis

**DOI:** 10.3389/fendo.2021.735954

**Published:** 2021-11-01

**Authors:** Yujie Shang, Huifang Zhou, Ruohan He, Wentian Lu

**Affiliations:** ^1^ Department of Gynecology, Affiliated Hospital of Nanjing University of Chinese Medicine, Nanjing, China; ^2^ The First School of Clinical Medicine, Nanjing University of Chinese Medicine, Nanjing, China; ^3^ Maternal and Child Hospital of Hubei Province, Tongji Medical College, Huazhong University of Science and Technology, Wuhan, China

**Keywords:** diet, polycystic ovary syndrome, fertility, reproductive endocrine, meta-analysis

## Abstract

**Objective:**

Diet has been reported as the first-line management of polycystic ovary syndrome (PCOS). However, the relationship between diet and fertility in PCOS is still controversial. This meta-analysis aimed to evaluate whether diet could promote reproductive health in women with PCOS while providing evidence-based nutrition advice for clinical practice.

**Methods:**

Seven databases, including Cochrane Central Register of Controlled Trials, PubMed, Embase, Web of Science, and some Chinese database, were searched up to January 31, 2021. Randomized controlled trials evaluating the effects of diet in women with PCOS were included. Based on a preregistered protocol (PROSPERO CRD42019140454), the systematic review was performed following the Preferred Reporting Items for Systematic Reviews and Meta-analyses (PRISMA) guidelines. Two reviewers made study selection, data extraction and bias assessment independently. Risk ratios and mean difference with 95% confidence intervals were assessed by a random-effects model. Statistical heterogeneity within comparisons was evaluated by Cochran’s Q test and quantified by the I-squared (*I^2^*) statistic.

**Results:**

Twenty RCTs with 1113 participants were included. Results showed diet significantly related to improved fertility outcomes (increasing clinical pregnancy, ovulation and menstrual regularity rate; reducing miscarriage rate), reproductive endocrine [increasing sex hormone-binding globulin (SHBG); decreasing Anti-Müllerian Hormone (AMH), free androgen index (FAI), total testosterone (T)] and clinical hyperandrogenism (hirsutism assessed by Ferriman-Gallwey score) in PCOS. Specifically, subgroup analyses indicated low-carbohydrate diets were superior in optimizing reproductive outcomes and calorie restriction was critical in ameliorating hyperandrogenism. Additionally, the positive effects were associated with the treatment duration. The longer the duration, the greater the improvement was.

**Conclusion:**

Overall, diet is an effective intervention for improving fertility health, thus professional and dynamic dietary advice should be offered to all PCOS patients, based on the changeable circumstances, personal needs and expectations of the individuals.

## 1 Introduction

Polycystic ovary syndrome (PCOS) characterized by irregular cycles, ovulatory dysfunction, hyperandrogenism and polycystic ovarian morphology (PCOM) is one of the most common endocrine disorders in women of reproductive age, and is prone to increased risks of complications such as diabetes, cardiovascular disease and endometrial cancer in the long term (1–3). The prevalence ranges from 6% to 21% depending on the population studied and diagnostic criteria used (4, 5). PCOS is associated with the risk of infertility and adverse pregnancy outcomes (6, 7). It has been reported as the most common cause of ovulatory dysfunction, accounting for 80% of women suffering from anovulatory infertility (8).

Hyperandrogenism and insulin resistance (IR) are the core etiologic and primary endocrine characteristics of PCOS, which interplay each other in the occurrence and development of the disease. Visceral adiposity, common in both obese and non-obese women, has been proved to amplify and worsen hyperandrogenism and IR, and this would induce abdominal adipose accumulation in turn, thus forming a vicious feedback cycle. The interactions among androgen, IR and obesity profoundly affects endocrine metabolism, leading to ovulation disorders, impaired potential development of ovum, and poor endometrial receptivity. With the increased rates of weight gain and prevalence of excess weight in women with PCOS (up to 88%) (9, 10), reproductive health is further exacerbated, which adversely affects the condition and poses a major public health challenge mandating both prevention and treatment.

For infertility patients with PCOS, the treatment principle is to optimize the health status at first before therapy. Given the association of obesity and insulin resistance in POCS, the role of diet in the PCOS management has become a focus in both reproductive and endocrine research in recent years. Emerging evidence has suggested that well-adjusted, balanced diets, such as the Dietary Approaches to Stop Hypertension (DASH) diet, the Mediterranean diet, low-carbohydrate diets and vegetarian diets are beneficial for ameliorating metabolic disorder and fertility, as well as preventing future related pregnancy complications (11–18). The International Evidence-based Guideline for the Assessment and Management of PCOS also emphasized the importance of diet in PCOS, and recommended diet and exercise as the first-line management for women with PCOS, mostly overweight and obese patients (19). Despite the general recommendations, there is a lack of specific clinical application, as patients with PCOS seem reluctant to follow (20) and they are not willing to adopt self-help methods (21). The main barrier is that PCOS patients have limited access to professional dietary treatment due to inadequate knowledge of current dietary care for this population. Effective, evidence-based dietary strategies for optimizing fertility in women with PCOS are essential. Previous meta-analyses mainly focus on the impact of pharmacological treatment or changes of lifestyle, exercise or single nutrient, and most of them pay attention to the endocrine and metabolism outcomes with few lectures evaluating the effects of diet on fertility in PCOS (22–24). Hence, it is warranted to define the effectiveness of diet in promoting reproductive health among women with PCOS, in order to provide appropriate dietary advice for clinical practice.

## 2 Materials and Methods

This systematic review was in accordance with Preferred Reporting Items for Systematic Reviews and Meta-analyses (PRISMA) (25) and has been registered in the International prospective register of systematic reviews (PROSPERO) under the number CRD42019140454.

### 2.1 Search Strategy

Databases such as the Cochrane Central Register of Controlled Trials (CENTRAL), PubMed, Embase, Web of Science, China National Knowledge Infrastructure (CNKI), VIP information database and Wanfang Data were searched from inception to January 31, 2021. We also checked reference lists and conference proceedings manually to obtain additional relevant data. No language or publication date restrictions. The details of the search strategy in PubMed are shown in **Supplemental Table 1**.

### 2.2 Study Selection

Studies which met the following inclusion criteria were included: (1) parallel controlled RCTs, (2) evaluating the effects of diet on fertility in women with a clear diagnosis of PCOS, such as the Rotterdam Consensus, the National Institutes of Health (NIH) diagnostic criteria, Androgen Excess and PCOS Society (AE–PCOS) Position Statement, as well as the China Medical Association diagnostic criteria (consisted of the following two aspects: a. Suspected PCOS: oligomenorrhoea or amenorrhoea or abnormal uterine bleeding is required, meanwhile accompanied by evidence of clinical or biochemical hyperandrogenism and/or PCOM; b. Confirmed PCOS: based on the suspected PCOS diagnosis conditions, it can be diagnosed by excluding other diseases that can cause hyperandrogenism), (3) studies with exercise/medication as a cointervention in both intervention/control arms were also considered. The primary outcomes referred to clinical pregnancy rate (defined as the presentence of intrauterine gestational sac with foetal heartbeats), miscarriage rate (defined as loss of intrauterine pregnancy before 20 completed weeks of gestation) and ovulation rate (determined by ultrasound or increased progesterone) (26); the secondary outcomes included menstrual regularity rate, Anti-Müllerian Hormone (AMH), free androgen index (FAI), sex hormone-binding globulin (SHBG), total testosterone (T), Ferriman-Gallwey score. All outcomes were measured before and after the observation.

The exclusion criteria were as follows: (1) quasi-randomized trials, cohort or case-control studies, reviews, meta-analyses, case reports, animal or cell experiments, (2) women with other causes for hyperandrogenism and abnormal ovulation, or any cardiovascular and cerebrovascular diseases, psychiatric, or neurological problems, (3) interventions referring to single dietary components (e.g., vitamins, calcium), and (4) studies with insufficient data and unreported target outcomes.

Titles and abstracts of all potential studies were scanned to eliminate duplicated and ineligible studies. When the information was inadequate to make a decision, we sought further details from the original authors. Any discrepancies were resolved by discussion or consensus with the corresponding author.

### 2.3 Data Extraction

Two authors extracted the predefined information from eligible studies independently. Additional information was further sought from the trials which appeared to be eligible but with unclear explanation of methodology or unsuitable data for meta-analysis. We cross-checked the data to minimize potential errors, and solved disagreements by discussion with the corresponding author. Information was collected from the included trials regarding the following aspects: (1) study characteristics, including first author, year of publication and location, (2) participants, including sample size and diagnostic criteria for PCOS, (3) interventions, including dietary protocols, frequency and duration of treatment, and (4) outcome data at baseline and follow-up.

### 2.4 Risk of Bias Assessment

Two authors assessed the risk of bias by the Cochrane Collaboration’s tool in eligible trials. Studies were deemed as low, unclear risk, or high bias based on the following domains: selection bias, performance bias, detection bias, attrition bias, reporting bias and other bias (27).

### 2.5 Data Synthesis

Review Manager 5.4 were applied for statistical analysis according to the Cochrane Handbook for Systematic Reviews of Interventions (28). *P* < 0.05 represented statistical significance. Results reported as binary variables were expressed as risk ratio (RR) with 95% confidence intervals. Data represented in continuous forms were pooled for meta-analysis as the mean difference (MD) with 95% confidence intervals if all studies reported the same scales. When data were reported on different methods or scales, the standardized mean difference (SMD) with 95% confidence intervals was calculated.

Due to inevitable clinical heterogeneity between studies, random-effects model was a more appropriate method to calculate summary effect measures. Statistical heterogeneity within comparisons was evaluated by Cochran’s Q test and quantified by the I-squared (*I^2^*) statistic. *I^2^* values < 40% might be important, 30%-60%were deemed moderate, 50% to 90% were deemed substantial, and 75% to 100% were deemed considerable heterogeneity (27). Regarding missing data which was unobtainable from the original investigators, the analysis was performed on an intention-to-treat (ITT) basis for primary outcomes (i.e. including all participants in analysis, in the groups to which they were randomised). All imputation were subjected to sensitivity analysis. We also conducted meta-regression and subgroup analyses to explain potential sources of heterogeneity between studies. Subgroup analyses based on the predefined factors, such as dietary patterns, intervention duration (< 3 months, 3-6 months or > 6 months), diagnostic criteria and calorie restriction were also conducted to explore mediator effects of dietary modification.

Sensitivity analyses were performed to examine the robustness of pooled estimates. When there were more than ten trials included in the analysis, the potential publication bias was investigated by Egger’s test and visual inspection of funnel plots.

## 3 Results

### 3.1 Study Selection

**Figure 1:**
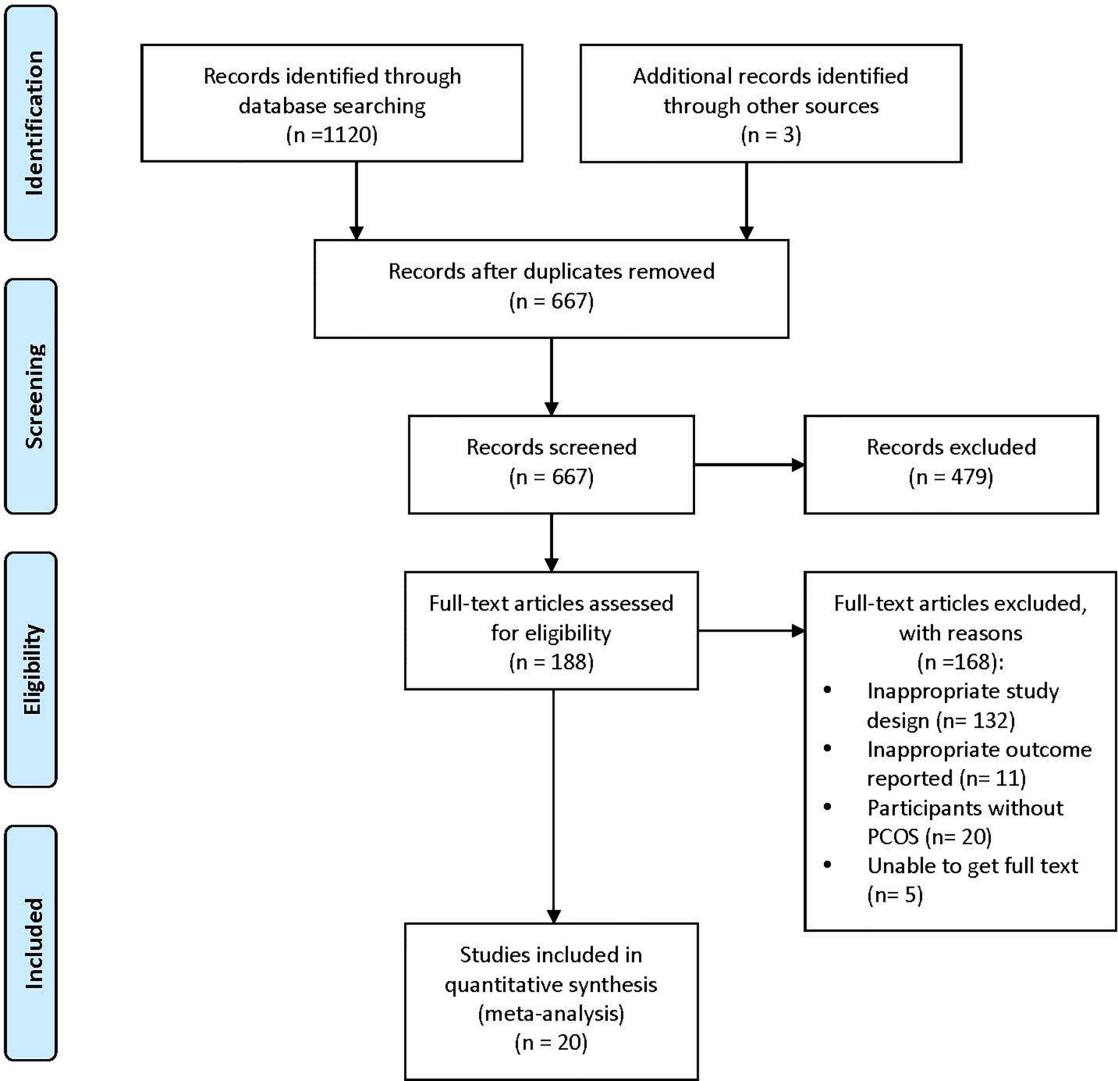
PRISMA flow diagram of study selection. PRISMA, Preferred Items for Systemic Reviews and Meta-analyses; PCOS, Polycystic Ovary Syndrome.

1123 studies were identified by the preliminary search. 456 records were removed due to duplication, and 667 studies were remained for further scanning of titles and abstracts. Among them, 479 items were excluded. We retrieved 188 full-text articles for detailed evaluation, and 168 trials were excluded for not meeting the inclusion criteria. Finally, 20 RCTs were included for meta-analysis. Details were shown in a PRISMA flow diagram (**Figure 1**).

### 3.2 Study Characteristics

The general characteristics of the included studies are outlined in **Table 1**. Except for the trial conducted by Gower et al. (crossover study) (34), all of them were parallel-design and single-canter RCTs conducted in China (40, 42–47), Iran (35, 36, 39, 41), the United States (30, 34), Australia (29, 32), the United Kingdom (31), Canada (48), Denmark (33), Egypt (37) and Mexico (38) between 2003 and 2020. The diagnosis of PCOS in the analysis could be divided into four categories: twelve trials under the Rotterdam Consensus (31, 33, 35, 36, 38–41, 43, 44, 46, 47), six trials following the NIH diagnostic criteria (29, 30, 32, 34, 37, 45), and the remaining two confirmed by the AE–PCOS (48) and China Medical Association (42) diagnostic criteria, respectively. Regarding dietary patterns, nine trials evaluated the low-carbohydrate diet (29, 30, 32, 33, 40, 42, 43, 46, 47); six trials respectively the low glycemic index/load diet (LGI/LGL) (31, 34, 37, 38, 45, 48); four trials evaluated the DASH diet (35, 36, 39, 41); and one trial evaluated the Mediterranean diet (44). The duration of diet ranged from one month to one year. Most of them had a medium duration (3-6 months), while four trials were within two months (30, 34–36) and one lasted for one year (42).

**Table 1:** Characteristics of trials included in the meta-analysis.

Author year (ref.)	Country	Diagnostic criteria	Sample size (n)	Weight changes (kg)	Type of diet	Control arm	Calorie restriction	Duration (month)	Outcomes
Moran et al. (2003) (29)	Australia	NIH	LCD: 23	LCD: -8.5 ± 1.1	LCD: CHO, 55%; P, 31%; F, 14%.	CON: CHO, 76%; P, 11%; F, 14%.	≤ 6000 kJ/d for the first 12 weeks in both intervention and control arms.	4	Primary: CPR, OR
			CON: 22	CON: -6.9 ± 0.8					Secondary: menstrual regularity rate
Stamets et al. (2004) (30)	USA	NIH	LCD: 17	LCD: -3.7 ± 1.9	LCD: CHO, 40%; P, 30%; F, 30%.	CON: CHO, 55%; P, 15%; F, 30%.	1000 kcal/d calorie deficit in both intervention and control arms.	1	Secondary: DHEAS, Ferriman-Gallwey score, T
			CON: 18	CON: -4.4 ± 1.5					
Atiomo et al. (2009) (31)	UK	Rotterdam	LGI: 6	NA	LGI diet provided by nutritionists.	CON: healthy eating approach.	600 kcal/d deficit in both intervention and control arms.	6	Secondary: number of menstrual cycles, SHBG, T
			CON: 5						
Moran et al. (2010) (32)	Australia	NIH	LCD: 24	LCD: -8.6 ± 20.7	LCD: CHO, 43%; P, 27%; F, 28%.	CON: CHO, 57%; P, 16%; F, 27%.	≤ 6000 kJ/d for the first 12 weeks in both intervention and control arms.	4	Secondary: FAI
			CON: 22	CON: -6.9 ± 18.2					
Sørensen et al. (2012) (33)	Denmark	Rotterdam	LCD: 29	LCD: -7.7 ± 20.6	LCD: CHO, 30%; P, 40%; F, 30%.	CON: CHO, 57%; P, 16%; F, 27%.	No calorie restriction.	6	Primary: CPR
			CON: 28	CON: -3.3 ± 13.73					Secondary: SHBG, T
Gower et al. (2013) (34)	USA	NIH	LGI: 30	NA	LGI: CHO, 41%; P, 19%; F, 40% (GI: 50).	CON: CHO, 55%; P, 18%; F, 27% (GI: 60).	No calorie restriction.	2	Secondary: FAI, SHBG, T
			CON: 30						
Asemi et al. (2014) (35)	Iran	Rotterdam	DASH: 27	DASH: -4.4 ± 2.7	DASH: CHO, 52%; P, 18%; F, 30%; rich in fruits, vegetables, whole grains, low-fat dairy products and low in saturated fats, cholesterol, refined grains, and sweets, with sodium was less than 2400 mg/day.	CON: CHO, 52%; P, 18%; F, 30%. The macronutrient composition was designed based on Iranian traditional dietary patterns.	350-700 kcal/d deficit depending on BMI in both intervention and control arms.	2	Primary: CPR
			CON: 27	CON: -1.5 ± 2.6					
Asemi et al. (2015) (36)	Iran	Rotterdam	DASH: 27	DASH: -3.6 ± 1.2	DASH: CHO, 52%; P, 18%; F, 30%; rich in fruits, vegetables, whole grains, low-fat dairy products, and low in saturated fats, cholesterol, refined grains and sweets, with sodium less than 2400 mg/day.	CON: CHO, 52%; P, 18%; F, 30%. The macronutrient composition was designed based on Iranian traditional dietary patterns.	350-700 kcal/d deficit depending on BMI in both intervention and control arms.	2	Primary: CPR
			CON: 27	CON: -1.3 ± 1.1					
Marzouk et al. (2015) (37)	Egypt	NIH	LGI: 30	LGI: -7.1 ± 12.1	LGI: CHO, 50%-55% (low GI); P, 15%-20%; F, 30% (calorie-restricted: 500 kcal/d).	CON: Follow the same healthy food of the intervention group without restriction in calories.	500 kcal/d deficit depending on BMI in both intervention and control arms.	6	Secondary: Ferriman-Gallwey score, number of menstrual cycles
			CON: 30	CON: -0.4 ± 12.8					
Sordia-Hernandez et al. (2016) (38)	Mexico	Rotterdam	LGI: 19	NA	LGI diet: CHO, 45-50%; P, 15-20%; F, 30-40; fiber: 20-35 g/d; GI: < 45.	CON: CHO, 45-50%; F, 30-40%; P, 15-20%; fiber20-35 g/d; GI: 50-75.	target calorie intake: 1200-1500 kcal/d, in both intervention and control arms.	3	Primary: OR
			CON: 18						
Azadi et al. (2017) (39)	Iran	Rotterdam	DASH: 30	DASH: -5.8 ± 1.9	DASH: CHO, 50%-55%; P, 15%-20% protein; F, 25%-30%; rich in fruits, vegetables, whole grains, low-fat dairy products, and low in saturated fats, cholesterol, refined grains and sweets, with sodium less than 2400 mg/day.	CON: CHO, 50%-55%; P, 15%-20%; F, 25%-30%.	350-700 kcal/d deficit depending on BMI in both intervention and control arms.	3	Primary: CPR
			CON: 30	CON: -4.3 ± 2.9					Secondary: FAI, SHBG, T
Fan et al. (2017) (40)	China	Rotterdam	LCD: 39	NA	LCD: give first place to low-carbohydrate, high-protein and low-calorie food with rich fiber. Vegetables and fruits are also essential. No alcohol and caffeine.	CON: advice on appropriate lifestyle.	No calorie restriction.	6	Primary: CPR
			CON: 39						
Foroozanfard et al. (2017) (41)	Iran	Rotterdam	DASH: 30	DASH: -4.3 ± 1.4	DASH: CHO, 52%-55%; P, 16%-18%; F, 30% fat; rich in fruits, vegetables, whole grains, low-fat dairy products, and low in saturated fats, cholesterol, refined grains and sweets, with sodium less than 2400 mg/day.	CON: CHO, 52%-55%; P, 6%-18%; F, 30% fat. The macronutrient composition was designed based on Iranian traditional dietary patterns.	350-700 kcal/d deficit depending on BMI in both intervention and control arms.	3	Primary: CPR
			CON: 30	CON: -3.2 ± 1.9					Secondary: AMH, FAI, SHBG, T
LI et al. (2017) (42)	China	CMA	LCD: 39	NA	LCD: CHO <30%; P ≥40%; F, 30%.	CON: nutritional counselling only.	No calorie restriction.	12	Primary: CPR, MR
			CON: 39						Secondary: menstrual regularity rate
Sun et al. (2017) (43)	China	Rotterdam	LCD: 32	LCD: -7.9 ± 8.9	a. LCD: weight loss period: about 50g/d carbohydrates; weight maintain period: <40% carbohydrates b. metformin: 1.5g/d	Metformin: 1.5g/d	No calorie restriction.	3	Primary: CPR
			CON: 32	CON: -4.7 ± 8.6					Secondary: menstrual regularity rate, FAI, SHBG, T
XU et al. (2017) (44)	China	Rotterdam	MedDiet: 20	MedDiet: -7.41 ± 5.0	Mediterranean diet: high intake of vegetables, legumes, fruits, nuts, cereals, and olive oil but a low intake of saturated lipids and meat, moderate intake of fish, low to moderate intake of dairy products, and regular but moderate intake of alcohol (usually wine).	CON: advice on daily health care.	No calorie restriction.	3	Primary: CPR
			CON: 20	CON: -2.2 ± 8.3					Secondary: menstrual regularity rate
YU et al. (2018a) (45)	China	NIH	LGL: 30	NA	a. LGL diet b. Ethinylestradiol and Cyproterone Acetate Tablets: ethinylestradiol 0.035 mg/d and cyproterone acetate 2 mg/d.	CON: Ethinylestradiol and Cyproterone Acetate Tablets (ethinylestradiol 0.035 mg/d and cyproterone acetate 2 mg/d).	No calorie restriction.	3	AMH, T
			CON: 30						
YU et al. (2018b) (46)	China	Rotterdam	LCD: 40	NA	a. LCD: CHO, 25%-30%; P, 40%-45%; F, 30%; rich in low-GI foods. b. fertility treatment.	CON: fertility treatment.	No calorie restriction.	3	Primary: CPR, OR
			CON: 30						Secondary: AMH
Zhu et al. (2019) (47)	China	Rotterdam	LCD: 40	NA	a. LCD: CHO, 50%; P, 20%; F, 30%. Rich in low-GI foods. b. fertility treatment.	CON: fertility treatment.	No calorie restriction.	3	Primary: CPR, MR, OR
			CON: 40						
Kazemi et al. (2020) (48)	Canada	AEPCOS	LGI: 30	LGI: 33	LGI: low-GI pulse-based diet; CHO, 52-55%; F, 30%; P, 16–18%; fiber: 33 g/d; GI: ∼35-40; GL: ∼70-100.	CON: CHO, 52-55%; P, 16-18%; F, 30%; fiber: 25 g/d; GI: ∼50-60; GL: ∼100-110.	No calorie restriction.	4	Secondary: menstrual cycle length, FAI, SHBG, T
			CON: 31	CON: 31					

AEPCOS, Androgen Excess and Polycystic Ovary Syndrome; AMH, Anti-Müllerian Hormone; BMI, Body mass index; CAM, China Medical Association diagnostic criteria (2011); CHO, carbohydrate; CPR, clinical pregnancy rate; CON, control; DHEAS, dehydroepiandrosterone sulfate; F, fat; FAI, free androgen index; GI, glycemic index; LCD, low-carbohydrate diet; LGL, low glycemic load; LGI, low glycemic diet; MedDiet, Mediterranean diet; MR, miscarriage rate; NIH, National Institutes of Health diagnostic criteria; NA, not available; OR, ovulation rate; P, protein; Rotterdam, European Society for Human Reproductive and Embryology/American Society for Reproductive Medicine diagnostic criteria; SHBG, sex hormone-binding globulin; T, testosterone; UK, the United Kingdom.

### 3.3 Risk of Bias Assessment

**Figure 2:**
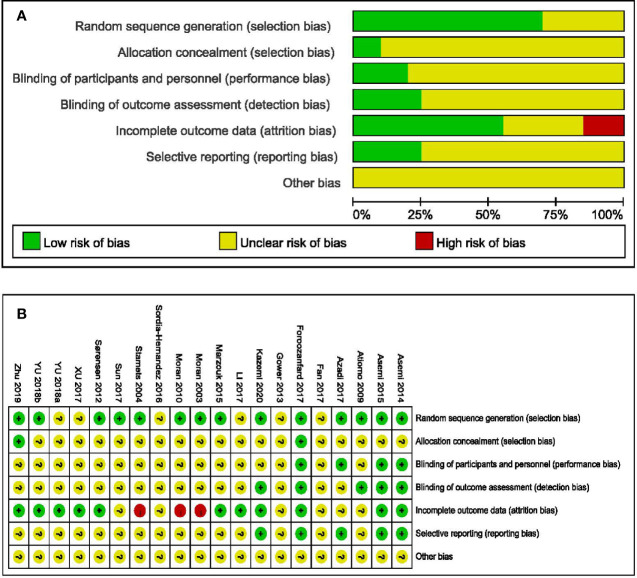
Assessments about risk-of-bias of included studies. **(A)** Risk of bias graph and **(B)** Risk of bias summary.

Fourteen studies provided data on randomization methods (29–33, 35–37, 39, 41, 43, 46–48), with two explaining the allocation concealment (41, 47). Blinding was only performed in four trials (35, 36, 39, 41), and were considered as low risk of bias. Four trials with participant-reported outcomes were judged as high risk, as the lack of participant blinding might introduce bias in these studies (29, 42–44). Five trials analyzed data on the ITT principle and were deemed as low risk (33, 35, 36, 41, 48). Five trials mentioning trial registration (35, 36, 39, 41, 48) were considered as low risk of reporting bias (**Figure 2**) (49).

### 3.4 Synthesis of Results

#### 3.4.1 Clinical Pregnancy Rate

**Figure 3:**
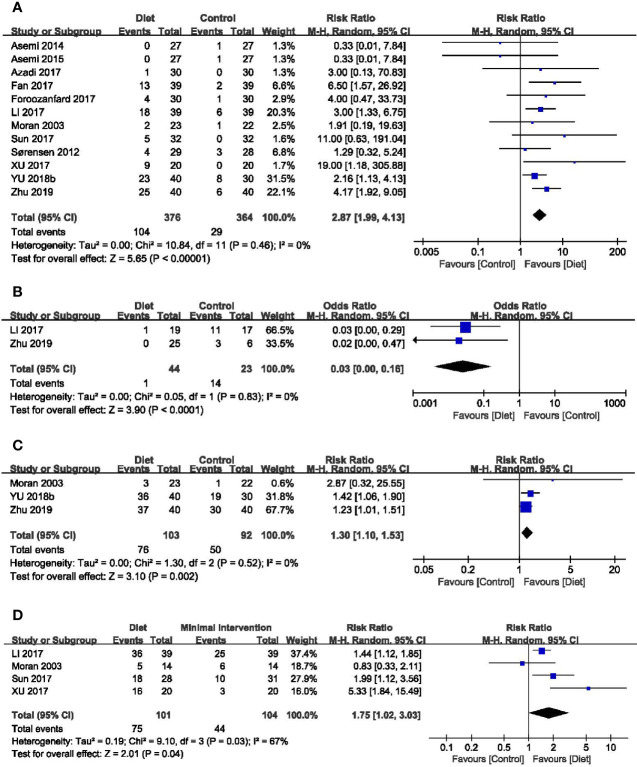
Forest plots of meta-analysis for **(A)** clinical pregnancy rate, **(B)** miscarriage rate, **(C)** ovulation rate, and **(D)** menstrual regularity rate.

Twelve trials (740 participants) reported the effect of diet on clinical pregnancy rate (29, 33, 35, 36, 39–44, 46, 47). The pooled data indicated a higher pregnancy rate in participants with dietary interventions (RR = 2.87, 95% CI: 1.99, 4.13; *P* < 0.00001), without between-grouped heterogeneity (*I^2^* = 0%) (**Figure 3A**). Subgroup analyses showed that both the Mediterranean diet and the low-carbohydrate diet were more beneficial to pregnancy, while the DASH diet had no advantages on the clinical pregnancy rate. In addition, the probability of becoming pregnant increased with the course of treatment, as it was non-significant when the duration was less than three months, while the effects became significant when the duration getting longer. Except for women diagnosed by NIH diagnostic criteria, diet was more effective for a better pregnancy rate among those according to Rotterdam Consensus and China Medical Association diagnostic criteria. No different effect was observed between groups when came to calorie restriction (**Table 2**).

**Table 2:** Effect estimates and heterogeneity of subgroup analysis for outcomes.

Subgroup	Trials (n)	Sample size (n)	Effect Estimate MD (95% CI)	*I²*	*P*
**Clinical pregnancy rate (%)**					
Diet type					
DASH diet	4	228	1.45 (0.36, 5.81)	0%	0.60
Low-Carbohydrate diet	7	473	2.92 (1.99, 4.27)	0%	< 0.00001
Mediterranean diet	1	40	19.00 (1.18, 305.88)	NA	0.04
Intervention duration					
< 3 months	2	108	0.33 (0.04, 3.11)	0%	0.33
3-6 months	9	541	2.68 (1.82, 3.95)	0%	< 0.00001
> 6 months	1	78	3.00 (1.33, 6.75)	NA	0.008
Diagnostic criteria					
NIH	1	45	1.91 (0.19, 19.63)	NA	0.53
Rotterdam	10	617	2.93 (1.74, 4.91)	16%	< 0.0001
CAM	1	78	3.00 (1.33, 6.75)	NA	0.008
Calorie restriction					
Yes	7	415	2.64 (1.37, 5.07)	0%	0.004
No	5	325	3.17 (1.70, 5.93)	37%	0.0003
**Ovulation rate (%)**					
Diagnostic criteria					
NIH	1	45	1.25 (0.45, 3.52)	NA	0.67
Rotterdam	2	150	1.29 (1.09, 1.52)	0%	0.002
Calorie restriction					
Yes	1	45	1.25 (0.45, 3.52)	NA	0.67
No	2	150	1.29 (1.09, 1.52)	0%	0.002
**Menstrual regularity rate (%)**					
Diet type					
Low-carbohydrate diet	3	165	1.47 (1.08, 1.99)	21%	0.0004
Mediterranean diet	1	40	5.33 (1.84, 15.49)	NA	0.002
Intervention duration					
3-6 months	3	128	2.03 (0.82, 5.03)	0%	0.13
> 6 months	1	78	1.44 (1.12, 1.85)	NA	0.005
Diagnostic criteria					
NIH	1	28	0.83 (0.33, 2.11)	NA	0.70
Rotterdam	2	100	2.99 (1.16, 7.70)	60%	0.02
CAM	1	78	1.44 (1.12, 1.85)	NA	0.005
Calorie restriction					
Yes	3	165	1.47 (1.08, 1.99)	21%	0.01
No	1	40	5.33 (1.84, 15.49)	NA	0.002
**AMH**					
Diagnostic criteria					
NIH	1	60	-2.45 (-4.96, 0.06)	NA	0.06
Rotterdam	2	130	-2.01 (-2.71, -1.31)	49%	< 0.00001
Calorie restriction					
Yes	1	60	-1.40 (-2.54, -0.26)	NA	0.02
No	2	130	-2.22 (-2.40, -2.04)	0%	< 0.00001
**FAI**					
Diet type					
DASH	2	115	-4.60 (-9.92, 0.71)	50%	0.09
Low-carbohydrate diet	2	87	-1.80 (-2.70, -0.90)	0%	< 0.0001
LGI/LGL diet	2	114	-0.13 (-1.21, 0.96)	0%	0.82
Intervention duration					
< 3 months	1	50	-0.50 (-2.65, 1.65)	NA	0.65
3-6 months	5	266	-1.74 (-3.18, -0.31)	65%	0.02
Diagnostic criteria					
NIH	2	78	-0.88 (-2.69, 0.92)	0%	0.34
Rotterdam	3	174	-2.51 (-4.17, -0.85)	49%	0.003
AEPCOS	1	64	0.00 (-1.26, 1.26)	NA	1.00
Calorie restriction					
Yes	4	202	-2.27 (-3.44, -1.09)	24%	0.0002
No	2	114	-0.13 (-1.21, 0.96)	0%	0.82
**SHBG (nmol/L)**					
Diet type					
DASH	2	115	10.09 (-1.42, 21.60)	76%	0.09
Low-carbohydrate diet	3	136	4.89 (-4.17, 13.94)	50%	0.29
LGI/LGL diet	2	75	5.43 (-1.68, 12.53)	0%	0.13
Intervention duration					
< 3 months	1	50	-4.00 (-17.94, 9.94)	NA	0.57
3-6 months	6	276	7.81 (4.50, 11.11)	31%	< 0.00001
Diagnostic criteria					
NIH	1	50	-4.00 (-17.94, 9.94)	NA	0.57
Rotterdam	5	212	7.89 (3.80, 11.98)	44%	0.0002
AEPCOS	1	64	7.10 (-1.14, 15.34)	NA	0.09
Calorie restriction					
Yes	4	185	8.16 (5.80, 10.53)	56%	< 0.00001
No	3	141	3.94 (-2.69, 10.58)	0%	0.24
**T (nmol/L)**					
Diet type					
DASH	2	115	-0.24 (-0.34, -0.14)	0%	< 0.00001
Low-carbohydrate diet	3	112	-0.02 (-0.52, 0.48)	73%	0.94
LGI/LGL diet	4	185	-0.20 (-0.52, 0.12)	39%	0.21
Intervention duration					
< 3 months	2	76	-0.22 (-0.77, 0.33)	34%	0.43
3-6 months	7	336	-0.22 (-0.35, -0.08)	50%	0.002
Diagnostic criteria					
NIH	3	136	-0.31 (-0.59, -0.03)	5%	0.03
Rotterdam	5	212	-0.23 (-0.38, -0.08)	52%	0.003
AEPCOS	1	64	0.00 (-0.29, 0.29)	NA	1.00
Calorie restriction					
Yes	5	211	-0.27 (-0.35, -0.19)	1%	< 0.00001
No	4	201	-0.13 (-0.34, 0.07)	63%	0.21

AEPCOS, Androgen Excess and Polycystic Ovary Syndrome; AMH, Anti-Müllerian Hormone; CAM, China Medical Association diagnostic criteria (2011); FAI, free androgen index; GI, glycemic index; LGL, low glycemic load; LGI, low glycemic diet; MedDiet, Mediterranean diet; NIH, National Institutes of Health diagnostic criteria; NA, not available; Rotterdam, European Society for Human Reproductive and Embryology/American Society for Reproductive Medicine diagnostic criteria; SHBG, sex hormone-binding globulin; T, testosterone.

#### 3.4.2 Miscarriage Rate

Two trials identified the miscarriage rate (42, 47). Overall analysis revealed that dietary interventions were superior with a lower miscarriage rate than the control (RR = 0.03, 95% CI: 0.00, 0.16; *P* < 0.0001; *I^2^* = 0%) (**Figure 3B**).

#### 3.4.3 Ovulation Rate

Four trials (232 participants) mentioned the ovulation rate (29, 38, 46, 47). The study of Sordia-Hernandez reported the results as the ratio of ovulatory cycles to all cycles during the treatment and in favor of the diet (24.6%, 14/57 *vs* 7.4%, 4/54) (38). A significant improvement was observed in the overall analyses of the other three trials when compared the diet groups with the minimal treatment (RR = 1.30, 95% CI: 1.10, 1.53; *P* = 0.002; *I^2^* = 0%) (**Figure 3C**). Results of subgroup analyses revealed that improvements in the ovulation rate were only evident in women diagnosed by Rotterdam and trials without calorie restriction (**Table 2**).

#### 3.4.4 Menstrual function

Seven trials (337 participants) assessed the menstrual function in both diet and control groups (29, 31, 37, 42–44, 48). Trial conducted by Kazemi mentioned the menstruation patterns, and no difference was noted between groups (48). The study of Marzouk (37) and Atiomo (31) reported the number of menstrual cycles during the treatment and the results were in favor of the diet (MD = 0.69, 95% CI: 0.08, 1.30; *P* = 0.03; *I^2^* = 0%) (**Supplemental Figure 1**) (49). The other four trials evaluated the menstrual regularity rate, and overall analyses found an advantage of dietary interventions in regulating menstruation (RR = 1.75, 95% CI: 1.02, 3.03; *P* = 0.04; *I^2^* = 67%) (**Figure 3D**). Subgroup analyses based on diet type, both LCD and Mediterranean diet led to more improvement than the control groups. Women adhering to dietary interventions for 3-6 months seemed to have no obvious advantages in adjusting menstrual condition, while those for 12 months showed the superiority. Similar to clinical pregnancy rate, significant effects of diet were observed in women diagnosed by Rotterdam Consensus and China Medical Association diagnostic criteria. Dietary therapy with or without calorie restriction seemed not to alter its benefits in the menstrual regularity (**Table 2**).

#### 3.4.5 Anti-Müllerian Hormone (AMH)

**Figure 4:**
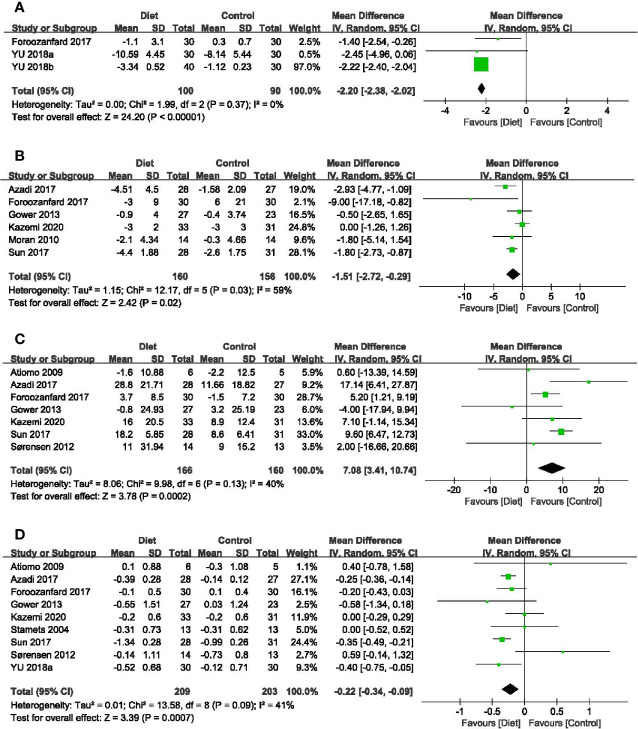
Forest plots of meta-analysis for **(A)** AMH, **(B)** FAI, **(C)** SHBG, and **(D)** T. AMH, Anti-Müllerian Hormone; FAI, free androgen index; SHBG, sex hormone-binding globulin; T, testosterone.

Three trials with 190 participants assessed the impact of diet on AMH levels (41, 45, 46). Meta-analysis revealed that dietary interventions resulted in a greater decrease in AMH (MD = -2.20 ug/L, 95% CI: -2.38, -2.02 ug/L; *P* < 0.00001), with no heterogeneity (*I^2^* = 0%) (**Figure 4A**). Subgroup analyses showed that diet led to more reduction in AMH concentrations among women diagnosed by the Rotterdam Consensus and the significant effects were found in both subsets, no matter with calorie restriction or not (**Table 2**).

#### 3.4.6 Free Androgen Index (FAI)

In total, six studies (316 participants) mentioned the changes in FAI (32, 34, 39, 41, 43, 48). Adherence to diet treatment was found to get more obvious improvement in the FAI (MD = -1.51, 95% CI: -2.72, -0.29; *P* = 0.02; *I^2^* = 59%) (**Figure 4B**). Results of subgroup analyses showed diet type, diagnostic criteria and calorie restriction might account for the heterogeneity. According to subgroup analyses between different dietary patterns, we found that low-carbohydrate diet could significantly affect FAI, while the DASH diet and LGI diet showed no advantages. Regarding diagnosis or energy intake, decreased FAI were more pronounced in women diagnosed by the Rotterdam Consensus or treated with calorie restriction. Besides, the effects might be associated with treatment duration time, as the reduction of long duration was more significant than that with a short one (**Table 2**).

#### 3.4.7 Ferriman-Gallwey Score

Two studies (86 participants) mentioned improved Ferriman-Gallwey score (30, 37). The results of meta-analysis suggested the superiority of diet in relieving clinical hyperandrogenism symptoms over the control (MD = -3.91, 95% CI: -5.87, -1.95; *P* < 0.0001; *I^2^* = 0%) (**Supplemental Figure 2**) **(** 49).

#### 3.4.8 Sex Hormone-Binding Globulin (SHBG)

In terms of SHBG, seven trials (326 participants) were included in the analysis (31, 33, 34, 39, 41, 43, 48). The significant difference was found in diet groups when compared with minimal intervention (MD = 7.08 nmol/L, 95% CI: 3.41, 10.74 nmol/L; *P* = 0.0002; *I^2^* = 40%) (**Figure 4C**). Results of subgroup analyses revealed that the improvement of diet in SHBG concentrations was evident only under the following conditions: diagnosed by the Rotterdam Consensus, an extended course and with calorie restriction (**Table 2**).

#### 3.4.9 Total Testosterone (T)

Nine studies (412 participants) examined the relationship between diet and change of testosterone levels (30, 31, 33, 34, 39, 41, 43, 45, 48). Meta-analysis showed that dietary interventions led to a greater decrease (MD = -0.22 nmol/L, 95% CI: -0.34, -0.09 nmol/L; *P* = 0.0007; *I^2^* = 41%) (**Figure 4D**). As to the subgroup analyses, the positive effects depended on the treatment duration. The longer the duration, the greater the improvement. Dietary intervention brought more reduction in T concentrations in patients taking the DASH diet or with calorie restriction. When grouped by the diagnostic criteria, the effects of diet were significant in trials followed the NIH diagnostic criteria and the Rotterdam Consensus (**Table 2**).

### 3.5 Meta-Regression Analyses

Meta-regression analyses were available only for clinical pregnancy rate. Factors, such as dietary patterns (regression coefficient β = 0.831; SE = 0.649; *P* = 0.241), treatment duration (regression coefficient β = 1.780; SE = 0.987; *P* = 0.114), diagnostic criteria (regression coefficient β = -0.937; SE = 0.908; *P* = 0.336) and calorie restriction (regression coefficient β = 0.726; SE = 0.591; *P* = 0.260) had no significant association with the study effect size. Meta-regression analyses were attempted to explain the heterogeneity among the studies, but inferences were limited by the paucity of available studies.

### 3.6 Sensitivity Analyses and Publication Bias

**Figure 5:**
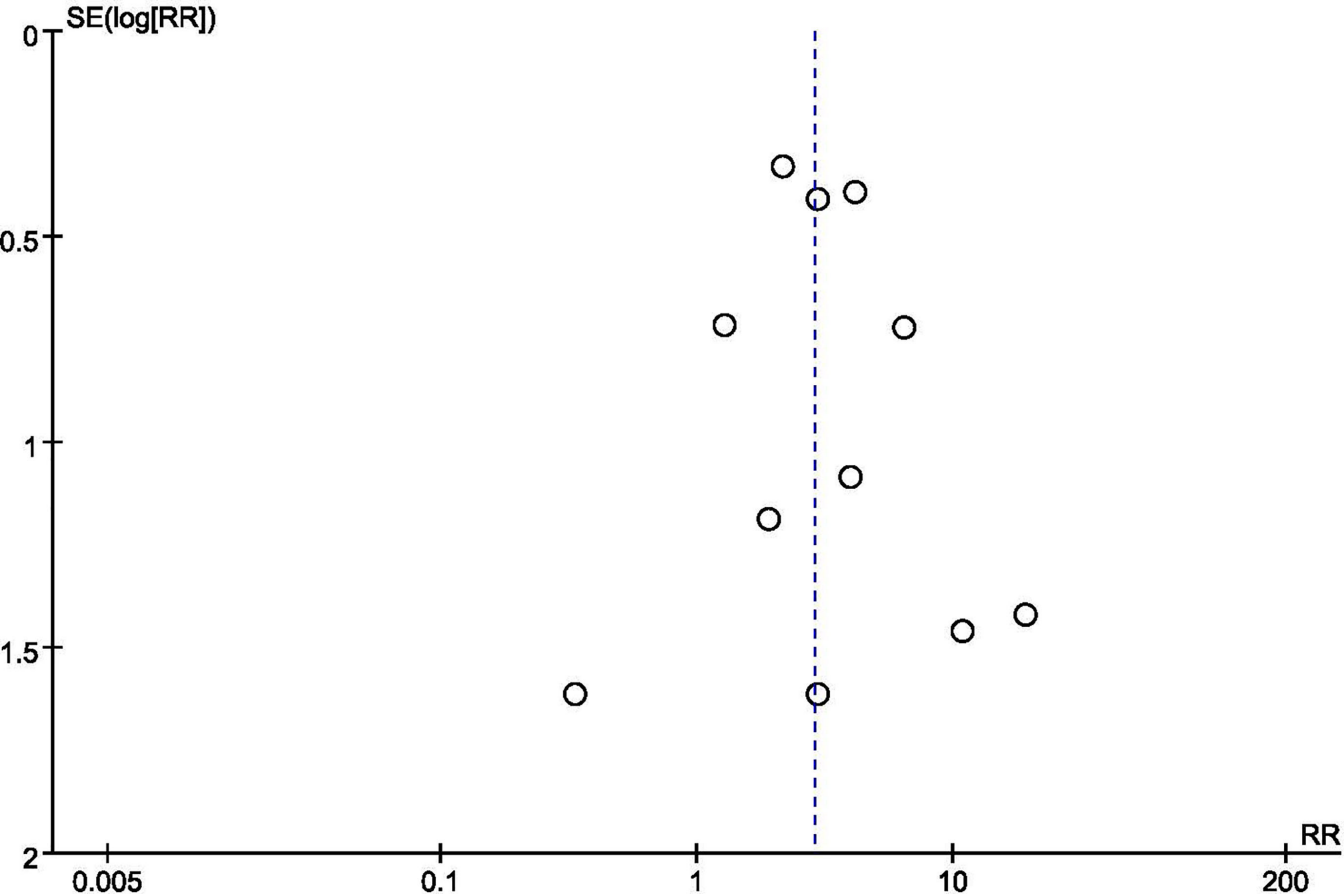
Funnel plot of clinical pregnancy rate.

We conducted sensitivity analyses by restricting studies to studies without a high risk of bias. When excluding trials deemed as high risk of bias, the overall estimates remained unchanged, indicating the majority of conclusions were stable and not affected by the low-quality trials. We also performed sensitivity analysis the imputation of primary outcomes. Similarly, when compared the pooled estimates of imputation and available data, no difference was noted. Given the limited number of studies (< 10), Egger’s test and the forest plot can be low-powered. Thus, we could only conduct tests on clinical pregnancy rate. The *P*-value of Egger’s test was 0.931, indicating no evidence of publication bias in this outcome. The funnel plot did not show major asymmetries (**Figure 5**).

## 4 Discussion

### 4.1 Principal Findings

In this systematic review and meta-analysis, the pooled data of 20 RCTs (1113 participants) showed that diet was not only associated with significantly improved fertility, but also mitigated hyperandrogenism in women with PCOS, which reiterated and extended those of previous reviews about the role of diet in endocrine, anthropometry and metabolism. In addition, these effects were associated with dietary patterns and treatment duration.

From the results of subgroup analyses, we found that low-carbohydrate diets tended to be better on improving pregnancy rate, reducing the risk of miscarriage and optimizing ovulation function. Our findings supported other notion in this topic. Several reports to date have showed that high-carbohydrate diets with a high glycemic index were associated with the increased risk of infertility concerning ovulatory disorders in apparently healthy women, while reducing carbohydrate consumption could influence the fertility and ovulatory function in turn (13, 50). Recently, there was evidence that the type of carbohydrate intake, such as low-glycemic index/load (LGI/LGL) food, was more important than the total amount received (51, 52). However, due to limited number of articles investigating the effectiveness of LGI/LGD diets on reproductive outcomes among women with PCOS, we were uncertain about its role in PCOS population

### 4.2 Comparison With Existing Studies

Consistent with previous research, we also found that calorie-restricted diets might be more salient in hyperandrogenism based on the subgroup analyses (53, 54). Hypocaloric diets could not only improve insulin sensitivity and regulate glycometabolism (55–57), but also advantageous for eliciting fast and significant weight loss, which exhibits a critical role in ameliorating PCOS phenotype. Weight reduction induced by calorie restriction is associated with reduced fat mass and preserved lean body mass (58), thus increasing the production of SHBG by the liver and reducing the levels of free testosterone (59, 60). However, dietary with energy limitation showed no effects in ovulation rate, and the improvement of clinical pregnancy rate, menstrual regularity rate and AMH level in women without calorie restriction were more obvious than those intaking fewer calories, which indicated that the benefits of diet might not just depend on weight loss, as not all PCOS patients with IR are overweight or obese and a higher incidence of IR have been reported in PCOS with normal weight (61, 62), suggesting that dietary management ought to go beyond weight loss. Of note, follicular development and ovulation require energy and energy requirements change during the menstrual cycle (63–65). Hence, it would be simplistic to claim for a beneficial effect of calorie restriction in all circumstances, since calorie restriction and consequent negative energy balance can also be harmful. Given this, different menstrual periods should also be considered during the calorie limitation. In our research, the favorable effects might also be associated with the treatment duration, as revealed in the subgroup analyses that the longer the duration, the greater the improvement was. Therefore, the diet treatment should be long term, dynamic and adapted to the changing circumstances, personal needs and expectations of the individual patient.

In our research, diet interventions were proved to increase the rate of decline of AMH, a well-recognized biomarker of ovarian reserve. Serum AMH concentration is higher in women with PCOS than in healthy women, which is related to severity of hyperandrogenism and oligo-anovulation (66, 67). A number of studies have reported that excess AMH could slow down initial follicular growth, decrease apoptosis of granulosa cells in small follicles with an anti-atretic effect, and cause follicular arrest in large antral follicles (68–70). Additionally, there is also a hypothesis that AMH appears to be able to exert its action at the hypothalamus and the pituitary level, which could either be at the origin of, or contribute to, the vicious circle of neuroendocrine and gonadal dysregulation encountered in PCOS (68). Therefore, the declined AMH levels might not only account for the reduced follicle excess of PCOM, but also line up with the elevated ovulation rate and ameliorative hyperandrogenism, thus improving the fertility outcomes.

The criteria used to diagnose PCOS were not uniform in this review, which might result in further clinical heterogeneity between studies. It has been reported that the overall prevalence of PCOS according to NIH criteria is 6%, while the pooled prevalence is 10% when applying the Rotterdam or AE-PCOS Society criteria. Studies in accordance with the NIH criteria, might narrow the phenotypic spectrum of PCOS and, thus limiting real PCOS population, as the morphology of polycystic ovarian is not considered as a diagnostic feature (71). However, the higher pooled prevalence estimates with the Rotterdam and AE-PCOS criteria is attributed to the inclusion of ovarian morphology and the ultrasound examination may provide false positive reports of PCOM (72). Besides, both oligo anovulation and PCOM are common in adolescent girls. Given this, the prevalence estimate might be exaggerated based on the Rotterdam criteria and people not suffering from PCOS truly might also be included. Therefore, due to the uncertainty surrounding the diagnosis of PCOS, and relative dearth of studies, we were unable to make conclusions concerning different diagnosis criteria.

### 4.3 Strengths and Limitations

Our research has unique strengths. To the best of our knowledge, this study is a frontier analysis to evaluate the role of diet on reproductive health in women with PCOS, in order to provide appropriate nutrition advice for clinical practice. We also performed detailed subgroup analysis based on different dietary patterns, treatment durations, diagnostic criteria, and whether energy was restricted, which may have significant impacts on the results. Since our research has been registered on PROSPERO, all the procedures were faithfully executed accordingly, as well as rigorous inclusion criteria, thus enhancing our results with more credibility and validity.

However, there were several limitations to be taken into consideration. Due to lack of livebirth rate, studies included were insufficient to address the role of diet on reproductive health comprehensively. Additionally, the evidence involved few countries and ethnic groups, which made the results difficult to be generalize. Besides, given the limited number of trials and small sample size in certain outcomes, the findings might be insufficient to ensure a significant difference.

### 4.4 Implications for Practice and Research

More well-designed studies are warranted to confirm the effects of dietary intervention on reproductive health in PCOS population. PCOS is a heterogeneous condition with different phenotypes. However, no included trials targeted a specific phenotype, which made the results difficult to generalize. Future work should focus on the relationship between reproductive health in particular phenotype and dietary intervention, thus investigating the effects accordingly. Sociodemographic disparities may have an impact on the effects of diet, such as economic status and educational attainment. Physicians should pay more attention to these factors mentioned above when designing RCTs and evaluate whether these issues would influence the observed outcomes and to what degree. Since that not all women with PCOS are overweight or obese, the impact of diet independent of weight loss is of great clinical interest. This review only compared diet with minimal intervention, future studies should expand the research scope and make comparisons with other commonly used pharmacological and surgical treatments or explore the possibility of combining interventions.

## 5 Conclusion

Findings of this review suggest that diet does benefit fertility health in women with PCOS. The higher adherence to low-carbohydrate diets, the higher possibility to get pregnant and regular menstruation. Additionally, it was calorie restriction that seemed to be more critical in ameliorating hyperandrogenism. Furthermore, the effects were associated with the course of treatment. Overall, diet is an effective intervention for improving fertility and reproductive health. More rigorous and large sample size RCTs are needed to confirm the effects and further explore the optimal dietary patterns.

## Data Availability Statement

The original contributions presented in the study are included in the article/**Supplementary Material**. Further inquiries can be directed to the corresponding author.

## Author Contributions

YS and HZ conceived and designed the review. YS and WL conducted the literature search. YS and RH performed the data extraction, quality assessment and statistical analysis. YS drafted the paper. HZ critically revised the manuscript. All authors contributed to the article and approved the submitted version.

## Funding

This study was supported by the National Natural Science Foundation of China (81973898) and Postgraduate Research & Practice Innovation Program of Jiangsu Province, China (SJCX21-0778).

## Conflict of Interest

The authors declare that the research was conducted in the absence of any commercial or financial relationships that could be construed as a potential conflict of interest.

## Publisher’s Note

All claims expressed in this article are solely those of the authors and do not necessarily represent those of their affiliated organizations, or those of the publisher, the editors and the reviewers. Any product that may be evaluated in this article, or claim that may be made by its manufacturer, is not guaranteed or endorsed by the publisher.
