# Supplementary material for: Dietary Modification for Reproductive Health in Women With Polycystic Ovary Syndrome: A Systematic Review and Meta-Analysis
Source: Front Endocrinol (Lausanne). 2021 Nov 1;12:735954. doi: 10.3389/fendo.2021.735954 (PMC8591222; doi:10.3389/fendo.2021.735954)
Supplement: Supplementary file 1 [file DataSheet_1.pdf]

Dietary modification for reproductive health in women with polycystic ovary syndrome: a systematic review and meta-analysis

Yujie Shang[1,2], Huifang Zhou[1,2*], Ruohan He[3], Wentian Lu[1,2]

[1]*Affiliated Hospital of Nanjing University of Chinese Medicine, Nanjing, China*

[2]*Nanjing University of Chinese Medicine, Nanjing,China*

[3]*Maternal and Child Hospital of Hubei Province, Tongji Medical College, Huazhong University of Science and Technology, Wuhan, China*

Content

Supplemental Table 1. Search strategy for PubMed (results from January 31, 2021). 3

Supplemental Figure 1. Forest plot of meta-analysis for changes in number of menstrual periods..5

Supplemental Figure 2. Forest plot of meta-analysis for Ferriman-Gallwey score...........................6

Supplemental Table 1 Search strategy for PubMed (results from January 31, 2021)

Search		Results
#1	diet[MeSH] OR diet[Title/Abstract] OR food[MeSH] OR food[Title/Abstract] OR feeding behavior[MeSH] OR dietary pattern[Title/Abstract] OR feeding pattern[Title/Abstract] OR eating behavior[Title/Abstract] OR food selection[Title/Abstract] OR dietary habit[Title/Abstract] OR dietary approach[Title/Abstract] OR food habit[Title/Abstract] OR eating habit[Title/Abstract] OR diet habit[Title/Abstract] OR lifestyle change[Title/Abstract]	1343493
#2	Polycystic Ovary Syndrome[MeSH] OR Ovary Syndrome, Polycystic[Title/Abstract] OR Syndrome, Polycystic Ovary[Title/Abstract] OR Stein-Leventhal Syndrome[Title/Abstract] OR Stein Leventhal Syndrome[Title/Abstract] OR Syndrome, Stein-Leventhal[Title/Abstract] OR Sclerocystic Ovarian Degeneration[Title/Abstract] OR Ovarian Degeneration, Sclerocystic[Title/Abstract] OR Sclerocystic Ovary Syndrome[Title/Abstract] OR Polycystic Ovarian Syndrome[Title/Abstract] OR Ovarian Syndrome, Polycystic[Title/Abstract] OR Polycystic Ovary Syndrome[Title/Abstract] OR Sclerocystic	19373

	Ovaries[Title/Abstract] OR Ovary, Sclerocystic[Title/Abstract] OR Sclerocystic Ovary[Title/Abstract] OR Polycystic ovary syndrome[Title/Abstract]	
#3	(clinical[Title/Abstract] AND trial[Title/Abstract]) OR clinical trials as topic[MeSH] OR clinical trial[Publication Type] OR random*[Title/Abstract] OR random allocation[MeSH]	1969449
#4	#1 AND #2 AND #3	395

	Diet			Control				Mean Difference	Mean Difference
Study or Subgroup	Mean	SD	Total	Mean	SD	Total	Weight	IV, Random, 95% CI	IV, Random, 95% CI
Atiomo 2009	2	1.5	6	1	0.5	5	22.8%	1.00 [-0.28, 2.28]	
Marzouk 2015	0.7	1.44	30	0.1	1.3	30	77.2%	0.60 [-0.09, 1.29]	
**Total (95% CI)**			**36**			**35**	**100.0%**	**0.69 [0.08, 1.30]**	

Heterogeneity: $Tau^2 = 0.00$; $Chi^2 = 0.29$, df = 1 (P = 0.59); $I^2 = 0\%$
Test for overall effect: Z = 2.22 (P = 0.03)

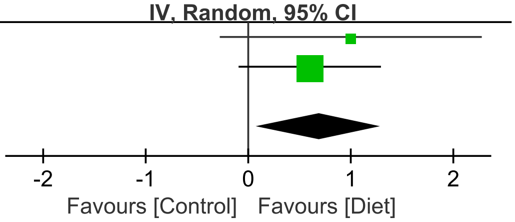

Supplemental Figure 1 Forest plot of meta-analysis for changes in number of menstrual periods.

	Diet			Control				Mean Difference
**Study or Subgroup**	**Mean**	**SD**	**Total**	**Mean**	**SD**	**Total**	**Weight**	**IV, Random, 95% CI**
Marzouk 2015	-4.2	5.31	30	0.7	5.88	30	47.9%	-4.90 [-7.74, -2.06]
Stamets 2004	-1	4	13	2	3	13	52.1%	-3.00 [-5.72, -0.28]
**Total (95% CI)**			**43**			**43**	**100.0%**	**-3.91 [-5.87, -1.95]**

Heterogeneity: Tau² = 0.00; Chi² = 0.90, df = 1 (P = 0.34); I² = 0%
Test for overall effect: Z = 3.91 (P < 0.0001)

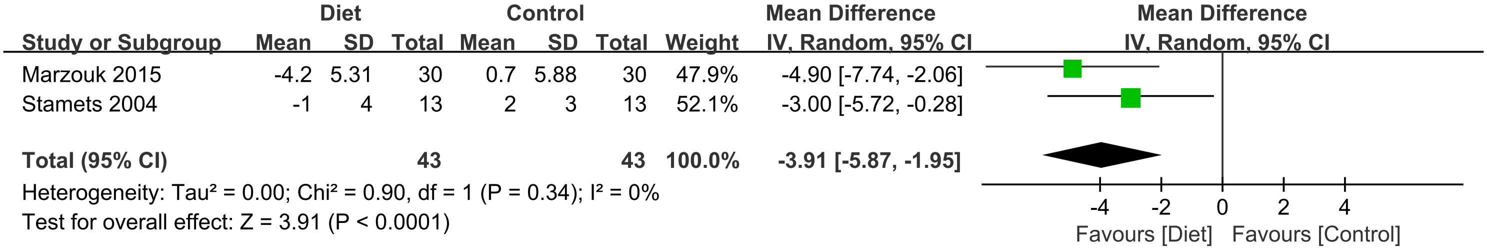

Supplemental Figure 1 Forest plot of meta-analysis for Ferriman-Gallwey score.
